# Maternal type 1collagen N-terminal telopeptide levels in severe hyperemesis gravidarum

**DOI:** 10.1186/s12884-018-2149-7

**Published:** 2018-12-20

**Authors:** E. Sahin, Y. Madendag, M. Eraslan Sahin, A. T. Tayyar, I. Col Madendag, M. Gozukucuk, C. Karakukcu, G. Acmaz, I. I. Muderris

**Affiliations:** 1 Department of Obstetrics and Gynecology, Health Sciences University, Sivas Sarkısla Government Hospital, Sivas, Turkey; 2 Department of Obstetrics and Gynecology, Health Sciences University, Kayseri Education and Research Hospital, Kayseri, Turkey; 3 Department of Obstetrics and Gynecology, Health Sciences University, Zeynep Kamil Maternity and Children’s Training and Research Hospital, İstanbul, Turkey; 4 Department of Biochemistry Clinic, Health Sciences University, Kayseri Education and Research Hospital, Kayseri, Turkey; 5 Department of Obstetrics and Gynecology, Erciyes University Faculty of Medicine, Kayseri, Turkey

**Keywords:** Hyperemesis gravidarum, Vitamin D deficiency, Urine type 1 collagen N-terminal telopeptide, Urine Ntx

## Abstract

**Background:**

Nausea and vomiting occur 50–90% during the first trimester of pregnancy. However, patients with hyperemesis gravidarum (HG) may be hospitalized at an incidence rate of 0.8–2% before the 20th week of gestational age. The symptoms generally start during the 5–6th gestational weeks, reaching the highest degree during the 9th week, and decline after the 16–20th weeks of gestation. Clinical findings are proportional to the severity of the disease and severe HG is characterized with dehydration, electrolyte imbalance, and nutritional deficiency as a result of vomiting.

**Methods:**

The study population consisted of two groups of pregnant volunteers at 5–12 weeks of gestation: a severe HG group and a control group. The HG severity was scored using the Pregnancy-Unique Quantification of Emesis (and nausea) (PUQE).The serum levels of the maternal Ca, parathyroid hormone (PTH), Na, K, blood urea nitrogen(BUN), creatinine, vitamin D(25OHD3), and the maternal urine NTx levels were compared between the groups.

**Results:**

In total, 40 volunteers were enrolled in this study: 20 healthy pregnant volunteers and 20 with severe HG. There were no statistically significant differences between the maternal characteristics. The first trimester weight loss of ≥5 kg was significantly higher in the severe HG group (*p* < 0.001), while the control group had a significantly higher sunlight exposure ratio than the severe HG group (*p* = 0.021). The urine NTx levels were significantly higher in the severe HG group (39.22 ± 11.68NTx/Cre) than in the control group(32.89 ± 8.33NTx/Cre) (*p* = 0.028).The serum Ca, PTH, Na, K, BUN, and creatinine levels were similar between the groups (*p* = 0.738, *p* = 0.886, *p* = 0.841, *p* = 0.957, *p* = 0.892, and *p* = 0.824, respectively). In the severe HG group, the serum 25OHD_3_ levels were significantly lower than in the control group (*p* < 0.001).

**Conclusions:**

The data from this study indicated that severe HG is associated with increased urine NTx levels. However, large-scale studies are required to understand the clinical significance of this finding, as well as the long-term consequences of elevated urine NTx levels and the underlying mechanisms.

**Trial registration:**

NCT02862496 Date of registration: 21/07/2016.

## Background

Nausea and vomiting occur 50–90% during the first trimester of pregnancy. However, patients with hyperemesis gravidarum (HG) may be hospitalized at an incidence rate of 0.8–2% before the 20th week of gestational age [1, 2]. The symptoms generally start during the 5–6th gestational weeks, reaching the highest degree during the 9th week, and decline after the 16–20th weeks of gestation [3]. Clinical findings are proportional to the severity of the disease and severe HG is characterized with dehydration, electrolyte imbalance, and nutritional deficiency as a result of vomiting [4–6]. Deficiencies in certain vitamins that work as catalyzers in the human body can lead to metabolic disorders.

Bone turnover is a dynamic and complex procedure, with bone resorption and bone formation being seen sequentially due to many different substances and factors. Biochemical markers, including protein and enzyme measurements, can be used to define bone resorption and formation [7]. Telopeptides are tiny protein particles found particularly in type I collagen that include 15–20 amino acids. Telopeptides bound to the ends of these amino acids are removed from the body through the urine as a result of collagen metabolism. During resorption, amino and carboxyl terminal fragments, called telopeptides, are bound to collagens with cross links and can be released into the circulation and excreted in the urine. Some recent studies have demonstrated that the type 1 collagen N-terminal telopeptide (NTx) level is more specific to bone tissue than any other resorption markers, showing more specificity if established earlier [8]. It can be used as a specific and stable indicator in the measurement of bone resorption.

It is well documented that sHG characterized by nutritional deficiency. We hypothesized that inadequate calcium and vitamin D intake may cause increased calcium mobilization from the maternal skeleton. Thus, in the present study, we aimed to determine the presence of maternal bone resorption in women with severe HG using the NTx levels in the maternal urine.

## Methods

### Study protocol

The present study was performed at Kayseri Education and Research Hospital in Kayseri, Turkey. The study was approved by the “Ethics Committee of Erciyes University” (decision number: 2016/345), which was carried out by the Declaration of Helsinki. All participants gave informed consent before the study.

The study population consisted of two groups of volunteers between 19 and 35 years of age who were between 5 and 12 weeks of gestation: an HG group and a healthy control group. HG was described as stated by the American College of Obstetricians and Gynecologists (ACOG) criteria: severe nausea and vomiting resulting in 5% weight loss (compared to pre-gestation) or vomiting more than three times per day and 3–5% weight loss with ketonuria. Those patients with the following other pathologies were excluded: gastroenteritis, pyelonephritis, bile duct diseases, hepatitis, urinary tract stones, hyperthyroidism, hyperparathyroidism, migraines and vestibular disease, and any other diseases causing NTx level changes (e.g., Paget’s disease and bone tumors) [3, 6]. In addition, those patients with any conditions causing osteoporosis or decreases in the bone mineral density were also excluded (smoking, celiac disease, inflammatory bowel disease, depression, pre-gestational diabetes, chronic kidney or liver disease, malignancy, and medication use, such as steroids, anticonvulsants, antiepileptics, heparin, or low molecular weight heparin) [9].

Pregnancy-Unique Quantification of Emesis (and nausea) (PUQE) questionnaire was used scoring the severity of each patient’s nausea and vomiting. The scores ≥13 points are classified as severe NVP/HG [10].

The blood samples were collected from the study population during the hospitalization of those patients diagnosed with severe HG. The blood samples from the volunteers in the control group were collected during their regular clinical visits.A5-mL blood sample was placed into a serum-separating tube to measure the serum Ca, PTH, Na, K, BUN, and creatinine levels. An additional 3-mL blood sample was placed into an EDTA tube to measure the serum 25OHD_3_ level. The blood samples were analyzed on the same day that they were obtained at the Kayseri Education and Research Hospital Biochemistry Laboratory. The 25OHD_3_ was measured using ultra high-performance liquid chromatography coupled with mass spectrometry (UHPLC-MS). While no consensus exists, we used a common clinical cut-off based on the Endocrine Society recommendations to categorize the 25OHD_3_ levels: deficiency < 25 nmol/L, insufficiency = 25 to < 50 nmol/L, and normal = 50–75 nmol/L [11].

NTx levels can be measured in serum and urine. In the present study we analyzed in urine because we aimed to measure to be non-invasively. In order to measure the urinary NTx level and urine creatinin**,** 10 cc of morning urine were collected following 12 h of fasting overnight the day after hospitalization. In control group morning urine samples were obtained the day after blood samples collected following 12 h of fasting overnight. Urine NTx levels vary throughout the day therefore, urine samples were analyzed from morning urine that NTx excretion was the most constant. The urine samples were immediately stored at − 80 °C, without blood contamination. The NTx levels were evaluated on the same day four months after the first urine sample was stored via an enzyme-linked immunosorbent assay by Cigdem Karakukcu. This was done to avoid any inconsistencies, and the samples were titrated with the urinary creatinine levels. All blood and urine samples were analyzed in Kayseri Education and Research Hospital Biochemistry clinic.

### Statistics

The Shapiro-Wilk’s test was used to test the normality assumption of the data. Levene’stest was used for the variance in homogeneity assumption. The values were expressed as the mean ± standard deviation, median (25–75^th^percentile), or n (%). The parametric comparisons were made using a t-test or z-test, and the nonparametric comparisons were made using the Mann-Whitney U test. All of the comparisons were made with the PASW Statistics 18 program, and a *p* value of < 0.05 was considered to be statistically significant.

The sample size was determined using a two-way analysis, and the reference values (means, standard deviations, and reference sample sizes) were taken from a study conducted by Shieh et al. [12]. It was estimated that at least 20 participants were required in each group to detect a clinically significant difference between the two groups when assuming a power of 80% to assess the primary hypothesis and a type I error of 0.05. Assuming a 5% dropout rate, we recruited 40 women (20 participants per group).

## Results

In total,40 pregnant women were enrolled in this study: 20 healthy pregnant women and 20with severe HG. The mean maternal age was 26.90 ± 2.36 years old in the control group and 26.95 ± 2.35 years old in the severe HG group (*p* = 0.947). The mean gestational age was 10.35 ± 1.23weeksin the control group and 10.30 ± 1.22 weeks in the severe HG group (*p* = 0.898). The pre-pregnancy body mass index (BMI) was 24.67 ± 1.27 in the control group and 24.76 ± 1.35 in the severe HG group (*p* = 0.829). The first trimester weight loss ≥5 kg was significantly higher in the severe HG group (*p* < 0.001), and the control group had a significantly higher sunlight exposure ratio than the severe HG group (*p* = 0.021). Overall, there were no statistically significant differences between the nulliparity, caffeine intake, exercise regularly, and family history of osteoporosis. The comparisons of other maternal characteristics between the groups are shown in Table 1.

**Table 1:** Comparison of maternal characteristics between the groups

	Severe HG group (*n* = 20)	Control group (*n* = 20)	*P* value
Maternal age (year)	26.95 ± 2.35	26.90 ± 2.36	0.947
Gestational weeks	10.30 ± 1.22	10.35 ± 1.23	0.898
Pre-pregnancy BMI (kg/m^2^)	24.76 ± 1.34	24.67 ± 1.27	0.829
First trimester weight loss ≥5 kg	19(95%)	0(0%)	< 0.001
Nulliparity	13(65%)	12(60%)	0.752
Exercise regularly	0(0%)	3(15%)	0.083
Family history of osteoporosis	3(15%)	3(15%)	1
Caffeine intake	0(0%)	0(0%)	1
Sunlight exposure	0 (0%)	4 (20%)	0.021

HG: hyperemesis gravidarum, BMI: body mass index

The Shapiro-Wilk’s test was used to test the normality assumption of the data. Levene’s test was used for the variance in homogeneity assumption. The values were expressed as the mean ± standard deviation, median (25–75th percentile), or n (%). The parametric comparisons were made using a t-test or z-test, and the nonparametric comparisons were made using the Mann-Whitney U test. All of the comparisons were made with the PASW Statistics 18 program, and a *p* value of < 0.05 was considered to be statistically significant

The biochemical parameters are compared in Table 2. The urine NTx levels were significantly higher in the HG group (39.22 ± 11.68 NTx/Cre nmolBCE/nmol)) when compared to the control group (32.89 ± 8.33NTx/Cre nmolBCE/nmol))(*p* = 0.028). Serum Ca, PTH, Na, K, BUN, and creatinine levels were similar between the two groups (*p* = 0.738, *p* = 0.886, *p* = 0.841, *p* = 0.957, *p* = 0.892, and *p* = 0.824, respectively). In the severe HG group, the serum 25OHD_3_ levels were significantly lower than those in the control group (*p* < 0.001).

**Table 2:** Comparison of biochemical parameters between the groups

	Severe HG group (*n* = 20)	Control group (*n* = 20)	*P* value
Serum BUN (5–15 mg/dl)	12.01 ± 1.09	11.96 ± 1.21	0.892
Serum creatinine (0.5–1.1 mg/dL)	0.91 ± 0.09	0.90 ± 0.08	0.824
Serum Na (136–145 nmol/L)	139(138–140.75)	139(138–140)	0.841
Serum K (3–5 mEq/L)	3.72 ± 0.13	3.72 ± 0.14	0.954
Corrected serum Ca	8.70(8.43–9.10)	8.75(8.40–9.05)	0.738
Serum PTH (10–65 pg/mL)	37.39 ± 12.93	36.85 ± 10.96	0.886
Serum 25OHD3 < 25 ng/mL (n%)	18(90%)	6(30%)	< 0.001
Urine NTx (NTx/Cre) (26–124 nmol BCE/nmol)	39.22 ± 11.68	31.89 ± 8.33	0.028

*HG* hyperemesis gravidarum, *BUN* blood urea nitrogen, *PTH* parathyroid hormone, *NTx* type 1 collagen N-terminal telopeptide

The Shapiro-Wilk’s test was used to test the normality assumption of the data. Levene’s test was used for the variance in homogeneity assumption. The values were expressed as the mean ± standard deviation, median (25–75th percentile), or n (%). The parametric comparisons were made using a t-test or z-test, and the nonparametric comparisons were made using the Mann-Whitney U test. All of the comparisons were made with the PASW Statistics 18 program, and a *p* value of < 0.05 was considered to be statistically significant

## Discussion

During pregnancy, the physiological upregulation of calcium is of vital importance for the mineralization and development of the fetal skeleton, with approximately 20–30 g of calcium being transferred from the mother to the fetus [13]. This calcium demand is regulated by various compensatory mechanisms, including increased renal reabsorption, increased intestinal dietary calcium absorption, and calcium mobilization from the maternal skeleton [14].The absorption of calcium in the maternal intestine doubles increasingly until the 12th week of gestation. The most important determinant of intestinal calcium absorption is vitamin D, which provides continuous maternal fetal calcium transfer [15, 16]. The healthy functioning of these compensatory mechanisms will ensure healthy maternal bone mineral density at the end of pregnancy and lactation. In presence of severe HG, due to inadequate intestinal compensatory mechanism, more calcium mobilized from the maternal skeleton. Thus, this clinical study aimed to investigate the maternal bone health in women with severe HG using the bone resorption marker NTx levels in the maternal urine.

Metabolic and nutritional disorders associated with the severity of the HG are likely to develop, and fetomaternal morbidity increases [17]. In the present study, we found that serum the 25OHD_3_ levels were significantly lower in the severe HG group when compared to the control group. These results could be explained by inadequate calcium and vitamin D intake due to nausea and vomiting. In the literature, more than 60% of these patients were determined to have thiamine, riboflavin, vitamin B6, vitamin A, retinal binding protein, and vitamin K deficiencies [18]. Chiossi et al. reported that HG patients developed Wernicke’s encephalopathy due to their thiamine deficiencies [19]. In addition, Corona G et al. reported the development of osmotic demyelination syndrome in these patients due to their metabolic disorders and hyponatremia [20]. In another study, Shigemi et al. reported that fetal coagulation developed in HG patients due to a vitamin K deficiency [21].

In the current study, we found that the severe HG group exhibited significantly greater weight loss when compared to the control group. In pregnancy, body fat increases lead to peripheral estrogen production. It has been demonstrated that the maternal estrogen levels, which have an anabolic effect on bone mineral density, increase during pregnancy [22, 23]. Both the compensatory mechanisms and increased estrogen production provide adequate calcium supplies for both the mother and the fetus [16]. In the presence of severe HG, our results indicated that certain regulatory mechanisms, such as intestinal calcium reabsorption via increased 25OHD_3_ were inadequate. In addition, in the presence of inadequate gestational weight gain, maternal estrogen production will inevitably be affected. Therefore, the present study hypothesized how this situation would affect the maternal bone health.

In the literature, it has been reported that pregnancy is associated with decreased bone mineral density, and that approximately 5% or more of the total maternal bone mass is mobilized [24, 25]. Currently there are some diagnostic procedures for evaluating the bone mineral density; however, their safe use in pregnant women has not yet been proven, and some are not recommended [26]. NTx which has a specific monotype amino acid sequence for bone tissue, can be used as a specific and stable indicator in the measurement of bone resorption [27]. In the present study, we found that the urine NTx levels were significantly higher in the severe HG group when compared to the control group. There are a few studies in the literature evaluating maternal bone health during pregnancy via bone resorption markers. For example, Ettinger et al. reported that a 1200-mg daily calcium carbonate supplementation during pregnancy reduced the NTx levels when compared to a placebo. In their study, the dietary calcium supplementation suppressed the maternal bone mobilization [28]. Similarly, Jonakiraman et al. showed that dietary calcium supplementation reduced the NTx levels in the third trimester [29]. In another study, Liu et al. reported that among 36 pregnant Chinese women with inadequate dietary calcium intakes, calcium supplementation significantly decreased the bone resorption markers [30].

Our findings can be interpreted as; Pregnancy may impact a woman’s bone mass, which is an important determinant of her subsequent risk of osteoporosis [31]. The possibility that the intrauterine programming of fetal bone growth may be an important determinant of osteoporosis is now being considered [32]. New evidence showed that maternal dietary deficiencies during pregnancy may be associated with a lower bone mass in her offspring later in life [33, 34]. Women’s with severe HG may have an increased risk for lower bone mass in offspring due to maternal dietary deficiency and increased maternal bone mobilization.

## Conclusions

The data from the present study indicated that severe HG is associated with increased urine NTx levels. Large-scale studies are required to understand the clinical importance of this finding, in addition to the long-term consequences of elevated urine NTx levels and the underlying mechanisms.

## Abbreviations

ACOG: American College of Obstetricians and Gynecologists
BUN: Blood urea nitrogen
HG: Hyperemesis gravidarum
NTx: type 1 collagen N-terminal telopeptide
PTH: Parathyroid hormone
PUQE: Pregnancy-Unique Quantification of Emesis
UHPLC-MS: Ultra high-performance liquid chromatography coupled with mass spectrometry
